# Tricuspid Valve Dysfunction: Role of Exercise Invasive Hemodynamic Assessment

**DOI:** 10.1016/j.jscai.2025.103613

**Published:** 2025-06-17

**Authors:** Ashish H. Shah, Triston Eastman, Robin A. Ducas, James W. Tam

**Affiliations:** a Section of Cardiology, Department of Internal Medicine, Max Rady Faculty of Health Sciences, University of Manitoba, Winnipeg, Manitoba, Canada; b Department of Internal Medicine, Max Rady Faculty of Health Sciences, University of Manitoba, Winnipeg, Manitoba, Canada

**Keywords:** exercise, invasive hemodynamics, tricuspid stenosis

A 34-year-old man, born with tetralogy of Fallot, underwent primary complete repair in 1988. In 2009, he underwent tricuspid valve (TV) replacement with a 29.0-mm Mosaic valve (Medtronic) to address severe tricuspid regurgitation. Approximately 4 years after surgery, he began experiencing exertional dyspnea, which progressed slowly over time. His symptoms were particularly evident when carrying weight in his hands, and his ability to walk briskly or run was limited to about 50.0 m. Although a β-blocker provided partial relief, he was unable to continue working as a cattle farmer due to his limitations.

Over the past 4 years, transthoracic echocardiograms consistently showed mean TV gradients of 6 mm Hg without significant changes (Figure 1A). These gradients were attributed to the relatively small size of the surgical valve and coexisting moderate tricuspid regurgitation, as regurgitant volume would increase diastolic flow through the valve, and resultant gradient (Supplemental Video 1). The right ventricular outflow tract was unobstructed, but moderately severe pulmonary regurgitation was present. The right ventricle appeared moderately enlarged with moderate systolic dysfunction. Despite these findings, his symptoms were deemed disproportionate to the echocardiographic data, prompting a decision to perform an exercise invasive hemodynamic assessment.

**Figure 1:**
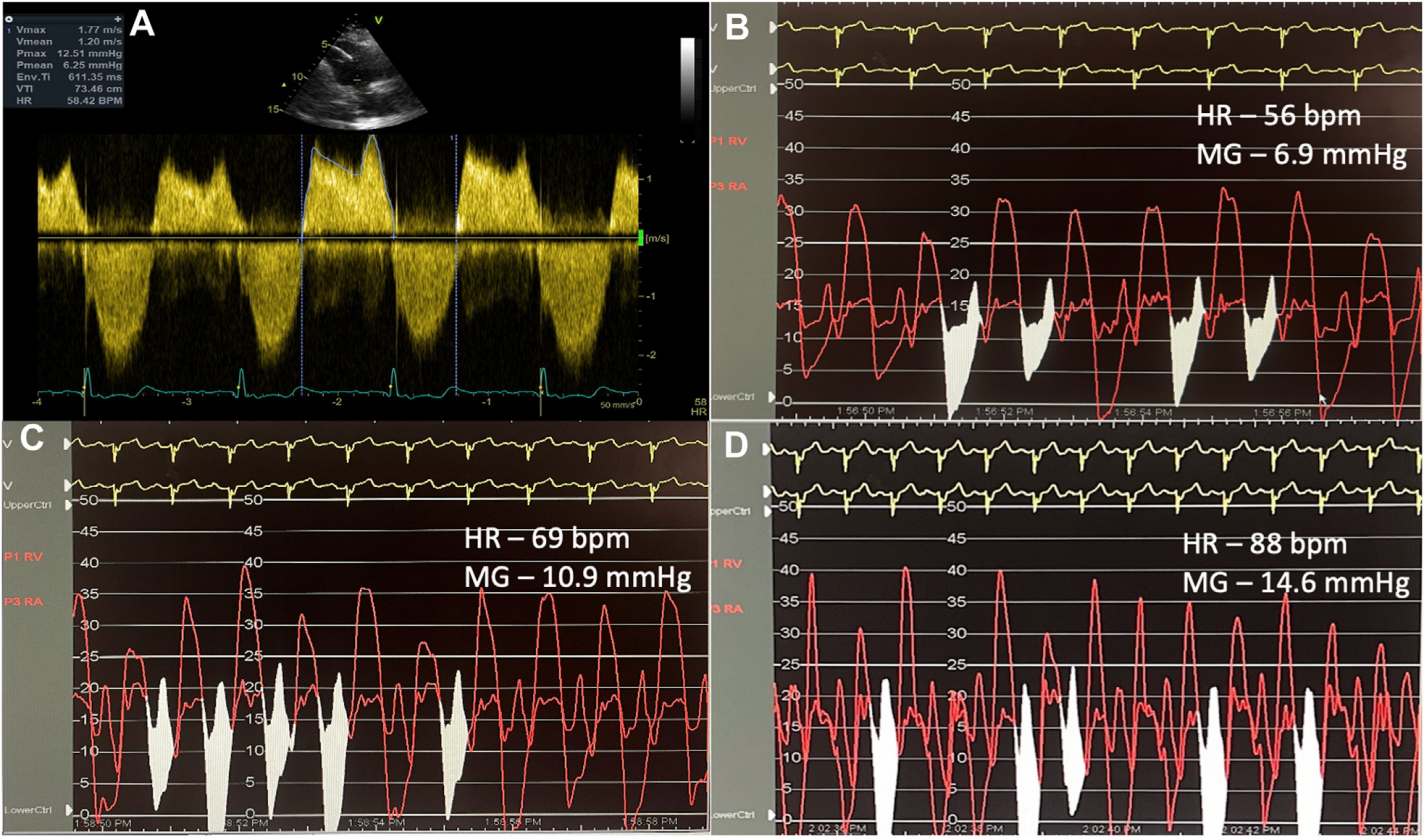
**Pressure gradients across the tricuspid valve at rest and with exercise.** (A) Doppler signal across tricuspid valve. Pressure gradient measurement across the tricuspid valve at rest (B), with incremental exercise (C), and peak exercise (D). HR, heart rate; MG, mean gradient; VTI, velocity time integral.

Vascular access was established via the right internal jugular vein using an 8F sheath. Hemodynamic measurements demonstrated mean right atrial pressure of 17 mm Hg (23/19/17 mm Hg), right ventricular pressure of 33/15 mm Hg, main pulmonary artery pressure of 24/11 mm Hg with a mean of 15 mm Hg, and mean capillary wedge pressure of 9 mm Hg. Simultaneous pressures across the TV were obtained using a 5F pigtail catheter into the right ventricle and the 8F sheath with tip into the right atrium. At rest, the mean gradient across the TV was 5.8 mm Hg at a heart rate of 54 beats per minute (Figure 1B). With exercise, including alternate leg raises and lifting 5 pounds in both hands, the mean gradient increased to 10.5 mm Hg (heart rate: 69 bpm) (Figure 1C), and subsequently to 12.7 mm Hg with a heart rate of 88 bpm (Figure 1D). A prominent “a” wave as a result of atria contracting against a relatively smaller TV was observed. At this point, the patient experienced his characteristic dyspnea symptoms and could not exercise further. Increased heart rate, such as with physical activity or atrial fibrillation, shortens the diastolic filling period and increases the gradient across the atrioventricular valve. Similar mean gradient across the tricuspid and mitral valves have different hemodynamic impact due to the variations in valve characteristics and differences in chamber pressure and compliance across the valve. For example, mean gradient of ≥5 mm Hg across TV describes severe stenosis, whereas for the mitral valve, it describes moderate stenosis only.^1^

Based on these findings, the patient underwent redo surgical TV replacement as a transcatheter valve-in-valve procedure would likely to have persisted with patient prosthesis mismatch.^2^ This case highlights the importance of exercise testing in assessing the hemodynamic impact of TV stenosis, enabling clinicians to better understand and address the true extent of valve dysfunction.^3^

## Pearls in Hemodynamics

• Resting hemodynamic assessmenet of atrioventricular valve stenosis may not unmask the true pathophysiology.

• Exercise invasive hemodynamic assessment should be considered, when patients experience exertional symptoms and there remains discrepancy between the symptoms and documented gradient at rest.

## Supplementary material

Supplemental Video 1

Supplemental Video 2 Color flow across the tricuspid valve.
